# Low Concentrations of Silver Nanoparticles in Biosolids Cause Adverse Ecosystem Responses under Realistic Field Scenario

**DOI:** 10.1371/journal.pone.0057189

**Published:** 2013-02-27

**Authors:** Benjamin P. Colman, Christina L. Arnaout, Sarah Anciaux, Claudia K. Gunsch, Michael F. Hochella, Bojeong Kim, Gregory V. Lowry, Bonnie M. McGill, Brian C. Reinsch, Curtis J. Richardson, Jason M. Unrine, Justin P. Wright, Liyan Yin, Emily S. Bernhardt

**Affiliations:** 1 Department of Biology, Duke University, Durham, United States of America,; 2 Civil and Environmental Engineering Department, Duke University, Durham, United States of America; 3 Department of Chemistry, Coe College, Cedar Rapids, United States of America; 4 Department of Geosciences, Virginia Tech, Blacksburg, United States of America; 5 Department of Civil and Environmental Engineering, Carnegie Mellon University, Pittsburgh, United States of America; 6 Duke Wetland Center, Nicholas School of the Environment, Duke University, Durham, United States of America; 7 University of Kentucky, Department of Plant and Soil Sciences, Lexington, United States of America; 8 Key Laboratory of Aquatic Botany and Watershed Ecology, Wuhan Botanical Garden, Chinese Academy of Sciences, Wuhan, People's Republic of China; 9 Center for the Environmental Implications of Nanotechnology (CEINT), Duke University, Durham, United States of America; University of Kansas, United States of America

## Abstract

A large fraction of engineered nanomaterials in consumer and commercial products will reach natural ecosystems. To date, research on the biological impacts of environmental nanomaterial exposures has largely focused on high-concentration exposures in mechanistic lab studies with single strains of model organisms. These results are difficult to extrapolate to ecosystems, where exposures will likely be at low-concentrations and which are inhabited by a diversity of organisms. Here we show adverse responses of plants and microorganisms in a replicated long-term terrestrial mesocosm field experiment following a single low dose of silver nanoparticles (0.14 mg Ag kg^−1^ soil) applied via a likely route of exposure, sewage biosolid application. While total aboveground plant biomass did not differ between treatments receiving biosolids, one plant species, *Microstegium vimeneum,* had 32 % less biomass in the Slurry+AgNP treatment relative to the Slurry only treatment. Microorganisms were also affected by AgNP treatment, which gave a significantly different community composition of bacteria in the Slurry+AgNPs as opposed to the Slurry treatment one day after addition as analyzed by T-RFLP analysis of 16S-rRNA genes. After eight days, N_2_O flux was 4.5 fold higher in the Slurry+AgNPs treatment than the Slurry treatment. After fifty days, community composition and N_2_O flux of the Slurry+AgNPs treatment converged with the Slurry. However, the soil microbial extracellular enzymes leucine amino peptidase and phosphatase had 52 and 27% lower activities, respectively, while microbial biomass was 35% lower than the Slurry. We also show that the magnitude of these responses was in all cases as large as or larger than the positive control, AgNO_3_, added at 4-fold the Ag concentration of the silver nanoparticles.

## Introduction

Engineered silver nanoparticles (AgNPs) are an emerging environmental contaminant of concern for regulators and consumer advocates because of their antimicrobial properties. AgNP production and incorporation into consumer products is increasing rapidly[1], [2], [3], and the majority of AgNPs released from consumer products are expected to enter terrestrial ecosystems through land-application of biosolids[4]. Given the critical role of microbial communities in organic matter and nutrient cycling in ecosystems, environmental exposures of AgNPs have the potential to alter ecosystem productivity and biogeochemistry[5].

Our knowledge of AgNP toxicity is largely drawn from controlled laboratory experiments with single strains of bacteria or fungi. These studies have shown that AgNP exposure leads to membrane damage, oxidative stress, and significant mortality [6], [7], [8], [9], [10]. AgNP toxicity is not limited to bacteria and fungi; recent studies have reported that AgNPs significantly reduced the growth of the annual grass, *Lolium multiflorum* [11], and reduced photosynthesis by the green alga, *Chlamydomonas reinhardtii* [*12*]. The mechanistic insights from these single-species experiments are vital to understanding AgNP impacts on organisms, but extrapolating from them to multi-species communities in complex environments is not practical. For example, the small number of laboratory AgNP exposure experiments conducted in environmental media (*i.e.*, soil, streamwater, sediment) with diverse native microbial communities have shown that AgNPs had no effects[13], [14], limited sublethal effects[15], or reduced effects in comparison to dissolved Ag[14], [16].

Additionally, the dominant route by which AgNPs will enter natural ecosystems is as aged particles through the land-application of wastewater treatment biosolids[4], not as Ag^0^ in freshly synthesized well dispersed nanoparticles. Biosolids are the processed or refined sewage sludge from wastewater treatment plants which are used in agricultural lands and rangelands as a nutritional soil additive. Recent work has shown that in biosolids, Ag is predominantly present as Ag_2_S, [17], [18] and sulfidation dramatically alters the properties of AgNPs including their surface charge, the ability to release Ag^+^ [19], and toxicity[20].

**Figure 1:**
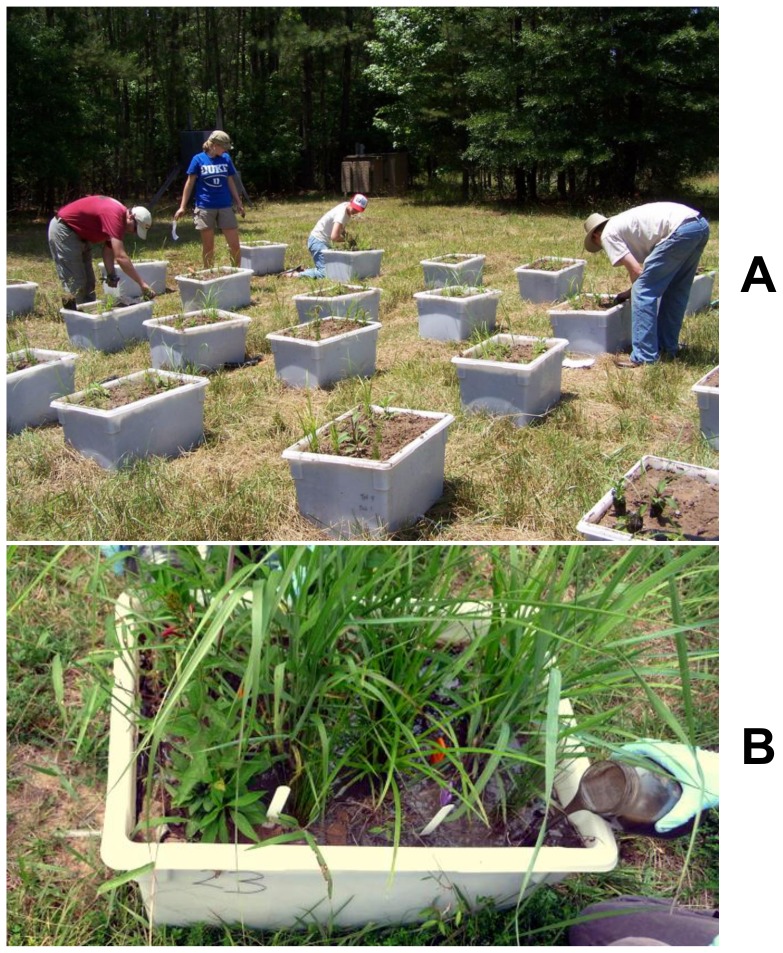
Terrestrial mesocosms in the Duke Forest, Durham, NC, USA. Mesocosms **A** on the day of planting, and **B** 63 days later (Day 0 of the experiment) mesocosms being amended with biosolid slurry

In this paper, we present a field experiment examining the ecosystem level impacts of AgNPs by using AgNP-dosed biosolids in outdoor mesocosms with diverse plant and microbial communities. We sought to answer three questions: 1) *What is the environmental fate of Ag under this exposure scenario?* 2) *How do realistic additions of AgNPs affect plant productivity, microbial community composition, and microbially-mediated biogeochemical cycling? 3) To what extent are the fate and biological impacts of AgNPs distinct in magnitude or direction from those of Ag^+^?* To address these questions we added biosolids alone, or biosolids amended with either polyvinylpyrrolidone-coated AgNPs or AgNO_3_ to a total of 24 replicate grassland mesocosms representative of abandoned agricultural fields found throughout the piedmont of the southern United States (Figure 1A, B).

### Setup and AgNP Addition

All mesocosms were filled with ∼81 kg of local floodplain soils that had been sieved (10 mm) to homogenize them and remove large rocks. Each mesocosm was planted with live plugs of three individuals of five commonly occurring plant species (*Carex lurida, Juncus effusus, Lobelia cardinalis, Microstegium vimineum,* and *Panicum virgatum)* on June 23, 2009 (Figure 1A). Plants were allowed to establish for 2 months prior to initiation of treatments. On August 25th, 2009 (Day 0), four experimental treatments were applied (Figure 1B): “Controls” received 1.5 L of deionized water; “Slurry” treatments received 1.5 L of biosolid slurry (200 g Class A biosolids mixed in deionized water, with a background of 1.5 mg Ag); “Slurry+AgNPs” received 1.5 L of biosolids containing 9.9 mg of Ag as AgNPs (total 11.4 mg Ag); and “Slurry+AgNO_3_” received 1.5 L of biosolid slurry containing 44 mg of Ag as AgNO_3_ to serve as a positive control (total 45.5 mg Ag). Assuming homogeneous mixing with the soil, these loading rates would give concentrations of 0.02, 0.14, and 0.56 mg Ag kg^−1^ soil in Slurry, Slurry+AgNPs, and Slurry+AgNO_3_ treatments, respectively. Each treatment was applied to six randomly-selected replicate mesocosms. This biosolid application rate of 870 g m^−2^ was based on US EPA guidelines [21], and Ag additions were based on a recent national analysis of biosolids contaminants[22]. Specifically, our AgNP treatment was at the 95% confidence interval of total Ag in biosolids, and our AgNO_3_ treatment was 4-fold higher to provide a positive control, while still remaining within the range of measured biosolid silver values.

**Figure 2:**
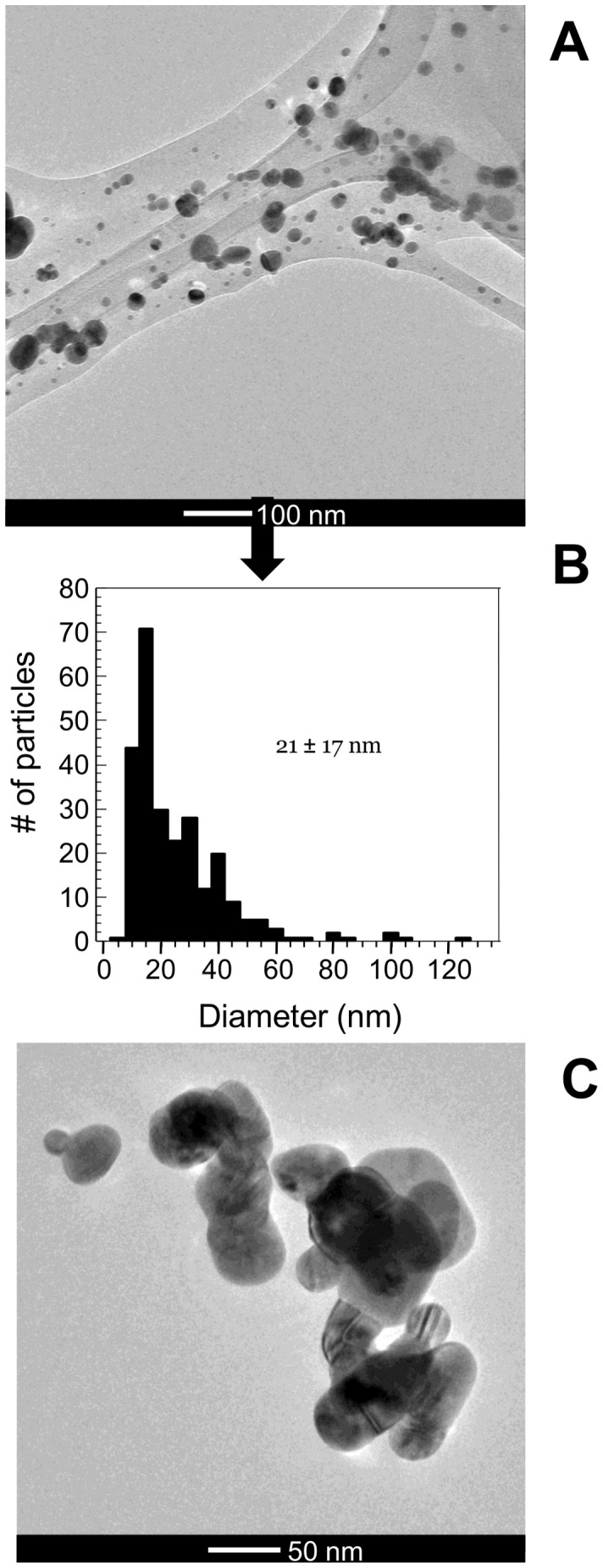
TEM characterization of particles and aggregates. TEM image **A** of a mixture of primary particles and particle aggregates **B** particle size distribution, and **C** detailed view of AgNP aggregate. (**A** and **B** are adapted from reference 23, and reproduced by permission of the Copyright Clearance Center on behalf of Elsevier)

For our AgNP treatment, we used a polyvinylpyrrolodine-coated AgNP powder that is representative of AgNPs that are commercially available for incorporation into consumer products (Nanoamorphous Materials, Los Alamos, USA). TEM analyses revealed particle diameters to be 21±17 nm (Figure 2A, B). Detailed characterization of these particles has been previously reported[23]. Briefly, particles in the stock suspension (see methods for stock suspension preparation) had a left skewed particle size distribution, were polydisperse, had a pH of 4.5, a zeta potential of −22.5 mV, and were about 10% Ag_2_O, and 90% Ag^0^ as measured by extended x-ray absorption fine structure [24].

To examine ecosystem level effects, we determined treatment differences in soil microbial community biomass, composition, and activity; plant biomass and photosynthesis; and trace gas fluxes from soils. Photosynthesis was measured on Days 8 and 30, while soil gas flux was measured on Days 8 and 50. Three replicate 1 cm diameter soil cores were collected for microbial community composition and microbial biomass on days 0 (before applying treatments), 1, and 50 of the experiment. On day 50 following gas flux measurements, all aboveground plant biomass was clipped at the soil surface and sorted by species. Following plant harvest, we collected three replicate 5 cm diameter soil cores, which were immediately separated in the field into depth classes (0–1, 1–5, 5–10 cm) and were subsequently used to determine root biomass, soil Ag content, microbial biomass, and extracellular enzyme activity.

Based on the results of previous laboratory experiments in environmental media [13], [14] we expected to see minimal effects from AgNPs in our mesocosm experiment compared to our AgNO_3_ positive control. Contrary to our expectations, microorganisms and plants showed predominantly adverse effects from the AgNP treatment, which in almost all cases were as large as or larger than the response to treatment with AgNO_3_ added at a four-fold higher concentration.

## Results and Discussion

### Silver in plants and soil

**Figure 3:**
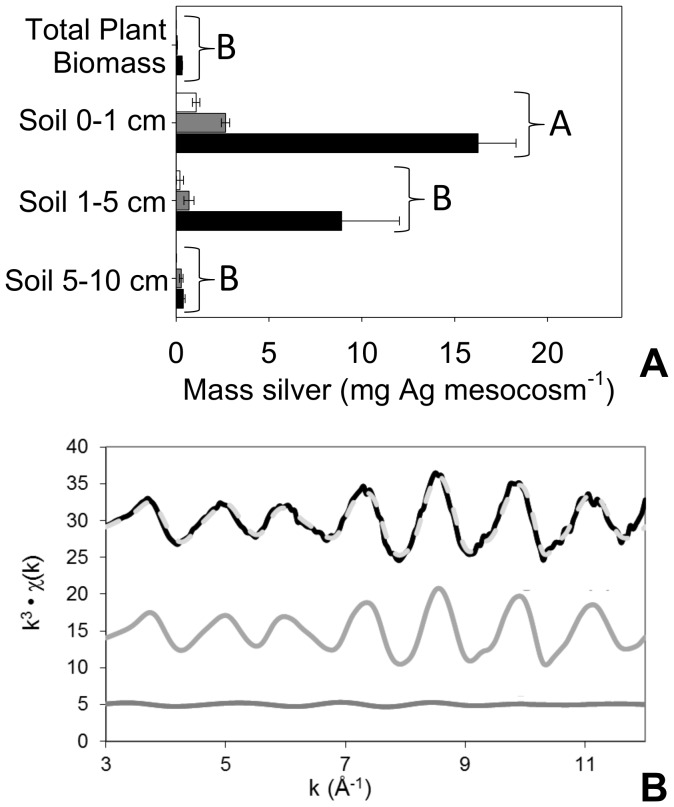
Silver fate in terrestrial mesocosms. **A** Recovery of silver by ecosystem compartment after 50 days exposure to biosolid Slurry (white bars), Slurry+AgNPs (gray bars), or Slurry + AgNO_3_ (black bars), and **B** EXAFS linear combination fit (k-space) of AgNPs after 15 minute exposure to biosolid slurry. In B, Lines indicate the data (black line), the linear combination fit (light gray dashed line), and the individual fit components Ag^0^ (gray line) and Ag_2_S (dark gray line) are shown, and represent 75±2% and 25±6% percent of the silver, respectively. The model R-factor  =  0.0672, chi^2^  =  86.64, and the reduced chi^2^  =  0.4867 (parameters describing goodness of fit of the model to the data). Error bars in panel **A** are standard errors of the mean (n = 6). Since all treatments showed the same pattern in ANOVA post-hoc testing, differences for each treatment within each ecosystem compartment were denoted with brackets with letters, where shared letters denote no significant difference at p<0.05 between ecosystem compartments within a treatment

Across all three biosolid treatments, Ag recovery averaged 58±25% (mean ± SD). This incomplete recovery is possibly due to a combination of factors including loss due to raindrop splash of biosolids, wind erosion of dessicated biosolid crust, movement of silver below 10 cm, and insect removal activity. Soils were the major sink for Ag in all cases (Figure 3A), which contained nearly 99% of the recovered Ag in all treatments. Aboveground and belowground plant tissues accumulated high concentrations of Ag in the Slurry+AgNPs and Slurry+AgNO_3_ treatments relative to Control (Table S1), but contained less than 1% of the recovered Ag (Figure 3A). Regardless of the initial form in which Ag was added (native Ag in Slurry, Slurry+AgNPs, or Slurry+AgNO_3_), concentrations and recoveries (Figure 3A) were highest in surface (0–1 cm) soils and decreased with depth to 10 cm where they were equivalent to concentrations measured in control treatment soils.

Given the low concentrations used in our experiment, direct observations of the physical and chemical properties of Ag recovered in our mesocosm soils and plants (*e.g.*, particle size, aggregation state, speciation) using techniques including transmission electron microscopy (TEM) and extended x-ray absorption fine structure (EXAFS) proved to be elusive. However, two lines of evidence suggest that AgNPs added to biosolids were rapidly transformed. Our first line of evidence comes from EXAFS data from a separate batch study of these same AgNPs and biosolids at a 10-fold higher concentration. The EXAFS data showed that after just fifteen minutes of exposure at 22 °C, 25% of the Ag was transformed into Ag_2_S (Figure 3B).The second line of evidence comes from the detection of Ag intimately associated with TiO_2_-NP aggregates[25] in our surface soils in the Slurry+AgNPs treatment (Figure S1), suggesting the potential for partial oxidation of Ag_2_S or AgNPs over the course of the experimental exposure, and that TiO_2_-NPs and other metal-oxide nanoparticles found in soils and biosolids may serve as a sink and/or carrier for Ag in the environment. Both lines of evidence are consistent with literature demonstrating oxidation and sulfidation of Ag in soils [20], [24] and biosolids [17], [18].

All five plant species accumulated significantly more Ag in aboveground tissues in the Slurry+AgNO_3_ treatment than in the Controls or Slurry (Table S1). Ag bioaccumulation tended to be lower for the AgNP treatment, yet all species except for *M. vimineum* and *L. cardinalis* had significantly higher tissue Ag concentration in the AgNP treatments than in Control or Slurry treatments. Interestingly, *M. vimineum* and *L. cardinalis* had the highest tissue Ag concentrations of any plants in both Ag treatments, but the high variability obscured statistical differences in the AgNP treatments. Some fraction of the silver associated with plants could have been due to rain associated spray, though the similar concentrations in the low creeping *M. vimineum* and the erect and tall *L. cardinalis* may suggest that it was not simply due to raindrop spray.

### AgNPs impacted above and belowground plant biomass

**Figure 4:**
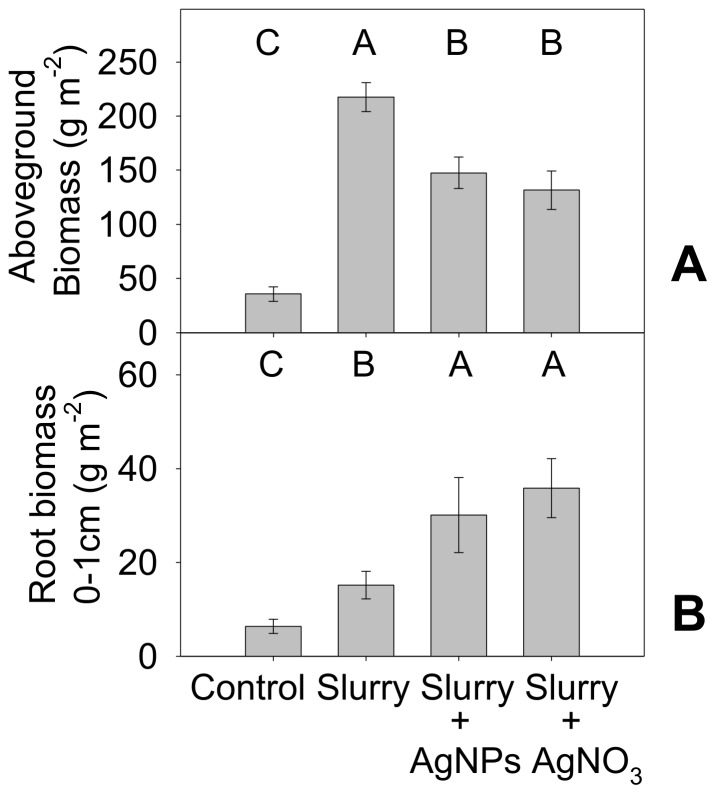
Mesocosm plant aboveground and belowground biomass affected by Ag. **A** Aboveground plant biomass of *Microstegium vimineum*, **B** root biomass in 0–1 cm soils. Error bars are standard error of the mean, and shared letters denote no significant difference at p<0.05 between treatments differences (n = 6)

Out of the 5 species we planted in the mesocosms, only *Microstegium vimineum* showed sensitivity to the AgNP treatment, growing 32% less aboveground biomass in the Slurry+AgNP treatment as opposed to the Slurry treatment (Figure 4A). While Ag toxicity may be tied to uptake in *M. vimineum*, *Lobelia cardinalis* had similar Ag concentrations in aboveground biomass, yet showed no evidence of toxicity. Thus uptake does not necessarily lead to growth inhibition, and it may be that different species of plants have different susceptibilities to Ag induced toxicity [26].

The Slurry+AgNP treatment also affected root biomass, in that there were significantly more roots in the shallowest (0–1 cm) soils of the Slurry+AgNP mesocosms than in the Slurry alone mesocosms (Figure 4B). However, total root biomass did not differ significantly between slurry treatments (Figure S2). Given the difficulty in separating roots out by species, it is not known whether these effects were due to a change in rooting depth and allocation of one, many, or all species.

### AgNPs impacted microbes

**Figure 5:**
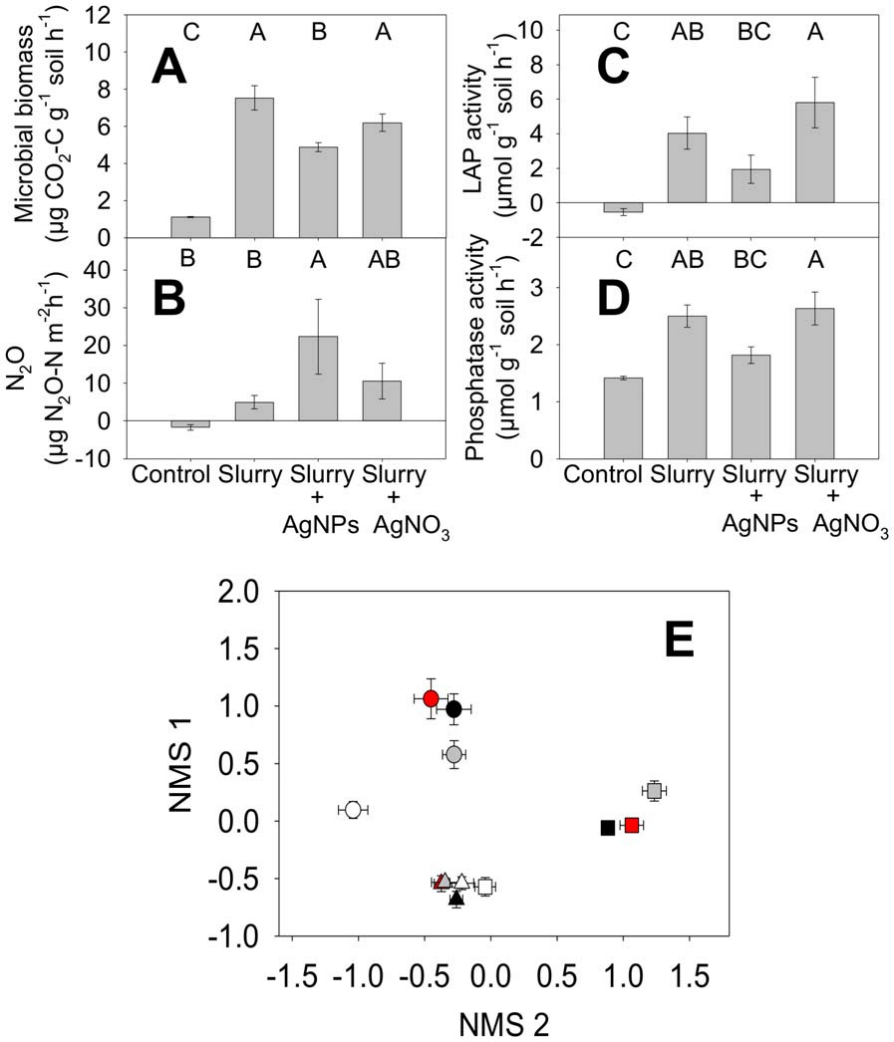
Microbial abundance, activity, and composition affected by Ag. **A** Microbial biomass in 0–1 cm soils on Day 50 of the experiment; **B** N_2_O flux from soil on day 8; **C** activity of the proteolytic extracellular enzyme leucine aminopeptidase (LAP), on day 50; **D** activity of the organophosphorous degrading enzyme phosphatase on day 50; **E** NMS ordination of bacterial community composition with day of experiment designated by shapes: Day 0 (triangles), Day 1 (squares), and 50 (circles); and treatment designated by colors: Control (white), Slurry (black), Slurry+AgNPs (gray), and Slurry+AgNO_3_ (red). All error bars are standard error of the mean, and shared letters denote no significant difference at p<0.05 between treatments in panels A–D (n = 6)

Microorganisms had broad sensitivity to the AgNP treatment, and we observed significant changes in their abundance, function, and community composition across our experiment. At the end of the experiment (Day 50), microbial biomass in 0–1 cm soils in the Slurry+AgNP treatment was significantly lower than the Slurry-only treatment (Figure 5A). Given that microbial biomass typically increases with increasing root biomass[27], and given the increase in root biomass in the 0–1 cm soils in the Slurry+AgNPs, this decrease in microbial biomass is opposite of our expectations. We hypothesize that the significant reductions in microbial biomass and changes in microbial activity were due to Ag^+^ released from partially sulfidized AgNPs[19], [20].

The most notable changes in microbial activity in the Slurry+AgNPs treatment were an increase in the flux of nitrous oxide (N_2_O) as measured on Day 8 (Figure 5B), and lower extracellular enzyme activity as compared to the Slurry treatment at Day 50 (Figure 5C, D). The N_2_O flux was 350% higher in the Slurry+AgNPs treatment than in the Slurry only treatment on Day 8, a dramatic increase given that N_2_O is both an important greenhouse gas with 296 times the global warming potential of CO_2_ [28], and N_2_O is also the dominant stratospheric ozone depleting substance[29]. We did not observe this difference in N_2_O flux on Day 50 (data not shown), however the activity of extracellular enzymes on Day 50 showed that differences persisted in microbial activity. These enzymes are often used as indicators of the microbial potential to decompose organic matter [30]. Both leucine aminopeptidase (degrades amines; Figure 5C) and phosphatase (degrades phospho-ester bonds; Figure 5D) decreased in concert with microbial biomass in the Slurry+AgNPs treatment compared to Slurry-only. Though neither difference was significant as measured by ANOVA post-hoc tests, both phosphatase activity and leucine aminopeptidase activity were tightly correlated to microbial biomass (r^2^ = 0.61, p<0.0001 and 0.56, p<0.0001 respectively). This suggests that these changes in activity may have been driven by changes in microbial abundance.

Changes in microbial biomass and activity were accompanied by changes in microbial community composition. We examined bacterial community composition using a genetic fingerprinting technique (terminal restriction fragment length polymorphism; T-RFLP) to assess the similarity in the bacterial 16S-rRNA genes from 0–1 cm soils sampled on Days 0, 1, and 50. Overall, after silver addition treatments differed in operational taxonomic unit richness (OTU; each fragment corresponded to an OTU, analogous to the species concept in other organisms) and bacterial community composition (Figure 5E). An examination of OTU richness showed that, prior to biosolid treatment, all treatments were statistically equivalent with an average of 150 ± 9 OTUs. On Day 1, the Slurry+AgNPs richness declined to 101 ± 22 OTUs, significantly lower than the control richness (142 ± 23 OTUs) or Slurry only treatment (136 ± 25 OTUs). By Day 50, the richness for all treatments was statistically similar again, with an average of 112.9 ± 17.1 OTUs. Our NMS ordination complemented our OTU richness trends, and showed that just one day post-dosing, the Slurry+AgNPs treatment community was significantly different from Slurry as seen in the increased distance between treatment averages (Fig 5E; Bray-Curtis ANOSIM value R = 0.3444, p<0.0117). By Day 50, the treatments were still divergent from their respective Day 0 and Day 1 values, but were no longer significantly different from each other.

### Effects of AgNO_3_ treatment ≤ AgNP treatment

While AgNO_3_ was added at a four-fold higher concentration to act as a positive control, its impact on plants and microbes was never greater than that of the AgNP treatment. In terms of observed plant effects, both the decrease in *M. vimineum* biomass and the increase in 0–1 cm root biomass relative to the Slurry treatment were of similar magnitude for both Slurry+AgNPs and Slurry+AgNO_3_ (Figure 4A, B). The magnitude of the AgNO_3_ treatment effect on microbial abundance, community composition, and function was also consistently equal to or less than the effects of AgNP treatment. Microbial biomass and enzyme activity on Day 50 were not significantly different between Slurry+AgNO_3_ and Slurry only (Figure 5A, C, D). Nitrous oxide fluxes on Day 8 were not significantly different between the Slurry and Slurry+AgNO_3_ treatments. Similar to Slurry+AgNPs, bacterial OTU richness declined (from 150 ± 9 down to 109 ± 11 OTUs), and bacterial community composition as indicated by T-RFLP was also different between Slurry and Slurry+AgNO_3_ on Day 1 (Bray-Curtis ANOSIM value R = 0.31, p<0.005).

The similar or stronger effects of AgNP treatment on activity, abundance, and composition of plants and microbes compared to AgNO_3_ treatment—even though AgNO_3_ was applied at a 4-fold higher total Ag concentration—was unexpected given earlier comparative studies[14]. We hypothesize that the AgNP treatment provided a slow release of Ag^+^ to the ecosystem, whereas Ag added as AgNO_3_ may have been immediately sequestered, perhaps as Ag_2_S or Ag-sulhydryl compounds [31]. This is consistent with the findings that partially sulfidized Ag NPs remained toxic to *Escherichia coli* in pure culture[20] and thus may remain bioavailable to plants and microbes [31].

Several factors were unresponsive to silver treatments. Total aboveground and belowground plant biomass did not differ significantly between the three slurry treatments (Figure S2), despite the significant decrease in *M. vimineum* biomass in both Ag treatments. Similarly, for the four plant species for which we measured photosynthesis (Day 8 and Day 30), we saw no evidence of a significant difference in photosynthesis in Slurry+AgNP or Slurry+AgNO_3_ as compared to Slurry (Table S2). The impacts of Ag exposure on microbes and plant roots were confined to the shallowest soil layers, with microbial and root biomass in soils below 1 cm showing no Ag effects (Figures S2 and S3). Although slurry additions led to increased soil nutrient concentrations (nitrate and phosphate; Figure S4), there were no differences between the three treatments that received biosolid slurry, despite changes in microbial abundance, community composition, and activity. There were also no significant differences in CO_2_ or CH_4_ flux from soils receiving Slurry, Slurry+AgNO_3_, or Slurry+AgNPs on either Day 8 or Day 50 (data not shown).

## Conclusions

An estimated 60% of the average 5.6 million tons of biosolids produced each year in the United States is land applied [32], and represents an important and understudied route of exposure of natural ecosystems to engineered nanoparticles. Our results show that biosolids amended with AgNPs at environmentally relevant concentrations and added to a diverse terrestrial ecosystem caused ecosystem-level impacts. Specifically, the AgNP treatment led to an increase in N_2_O fluxes, changes in microbial community composition, biomass, and extracellular enzyme activity, as well as species specific effects on aboveground plant biomass (*i.e.*, on *M. vimineum*). Moreover, for all of these parameters, the effect of the AgNP treatment was as large as or larger than that of the treatment with AgNO_3_ added at a four-fold higher concentration, though whether this is due to differences in bioavailability of AgNPs and AgNO_3_ when added to biosolids, saturated bioavailability of the AgNO_3_, or some other factor is beyond the scope of this experiment. Importantly, given that this experiment used a one-time application of biosolids, these impacts could be enhanced by repeated applications, with further changes expected in microbial and plant community composition and functioning in the ecosystem.

Our results also suggest that while AgNPs may be transformed in biosolids through oxidation and sulfidation, they still had an impact on plants and microbes. Whether these impacts were through direct or indirect plant or microbially mediated mechanisms remains unexplored. We have also demonstrated the potential for several species of plants to take up Ag from AgNPs in soils, though the extent to which different plant species accumulated Ag varied greatly. Uptake and incorporation of Ag into plant biomass suggests the potential for trophic transfer of Ag. Future studies are aimed at identifying the bioavailable forms of Ag and on the speciation of Ag taken up into plants.

## Materials and Methods

### Soils

The soils used in this experiment were surface mineral soils from the floodplain of the Sandy Creek Restoration in Durham, NC[33], which is located in the Duke Forest Teaching and Research laboratory. Soils are of the Cartecay series (Coarse-loamy, mixed, semiactive, nonacid, thermic Aquic Udifluvents) and Chewacala series (Fine-loamy, mixed, active, thermic Fluvaquentic Dystrudepts) and had 63.5% sand, 10.5% silt, 26 % clay, and 1.8% loss on ignition. Mesocosms were established in the Duke Forest by first screening soils for materials larger than 10 mm, and then adding ∼81 kg of soil to 21.5 gallon polyethylene tubs (Rubbermaid, Wooster, USA) equipped with drains at 10 cm and at the bottom.

### Plants

Mesocosms were planted on June 23, 2009 with fifteen plants (three replicates from each of five species) placed in random order in fifteen of the sixteen slots in a 4 × 4 grid. Plants included four species of plants native to NC meadows (*Carex lurida,* sedge; *Juncus effusus*, rush; *Lobelia cardinalis*, forb; and *Panicum virgatum,* grass; purchased from Mellow Marsh Farm, Silk Hope, USA), as well as the non-native invasive C4 grass, *Microstegium vimineum*, collected from the Duke Forest on June 23, 2009. For collection of *M. vimineum*, no specific permits were required, and permission for collection was granted by Judson Edeburn, Duke Forest Resource Manager. Plants were watered daily with groundwater until they were well established. The mesocosms were weeded twice before the experimental treatments were applied to keep non-target plants to a minimum. Non-target plants were collected when mesocosms were sampled and were grouped together as “Others”.

### Biosolids

The biosolids used in these experiments were rated Class A EQ, and were obtained from the South Cary Water Reclamation Facility (Apex, USA) on July 15^th^, 2009 as dried pellets for use as fertilizer (7.47 % moisture) with total N reported as 7.5%. The addition rate was calculated to meet plant N demands during the 8 weeks of the experiment using established guidelines[21] adapted for our 0.23 m^2^ mesocosms.

These biosolid pellets were used for their ease of transport, storage, handling, and homogeneity, but they are not the typical form of biosolids that are land-applied in many agroecosystems and rangelands. They are dried, which decreases their volume. They are sold to agricultural users, landscapers, and consumers for use in dried and pelletized form [34]. To make a slurry from these biosolids, 200 g of dried pellets were first rehydrated with 750 ml of water and homogenized using an immersion blender (KitchenAid, St. Joseph, USA) on high for three minutes. The slurry then sat for 2 minutes to allow the immersion blender to cool and the pellets to soften. Pellets were then homogenized for another 3 minutes and the volume was brought to 1.5L with deionized water.

### Silver nanoparticles and dosing concentrations

Instead of freshly synthesized particles, we chose to use a commercially available AgNP powder representative of what is available for incorporation into consumer products (Nanoamorphous Materials, Los Alamos, USA). Particles were purchased as a dry powder, and suspended to make a 250 mg Ag L^−1^ suspension in deionized water by sonicating them for 10 minutes at 100W with a Sonicator 4000 equipped with a ½ inch diameter flat titanium tip (Misonix, QSonica LLC, Newton, USA). Temperature was controlled by placing the beaker used to make the suspension in an ice-water bath. Particles in suspension had diameters of 21±17 nm as measured by TEM. Detailed information on particle characterization has been previously published [23]. For the positive control, we used AgNO_3_ (SigmaAldrich, St. Louis, USA).

Additions of Ag were chosen to fall within the range of concentrations of Ag measured in the United States Environmental Protection Agency's Targeted National Sewage Sludge Survey (TNSSS)[22], which reported concentrations between 2 and 195 mg Ag kg^−1^, and a mean ± SD of 20±22 mg Ag kg^−1^, excluding one outlier at 856 mg Ag kg^−1^. For AgNPs, we added 9.9 mg Ag per mesocosm of AgNPs in addition to the 1.5 mg Ag in the 200 g of biosolids (57 mg Ag kg^−1^ biosolids). This rate of addition represents the top end of the 95% confidence interval of the TNSSS. With the AgNO_3_ treatment, we wanted to have a positive control with a better documented toxin, and so we added 44 mg Ag as AgNO_3_ per mesocosm in addition to the 1.5 mg Ag in the 200 g of biosolids (228 mg Ag kg^−1^ biosolid), which is twice the 99% confidence interval of the TNSSS [22], but lower than the highest value detected in the TNSSS.

### Treatments

For the mesocosm experiment, manipulations were done on August 25, 2009. There were six replicates of each of four treatments, which were randomly assigned to each of the 24 mesocosms: “Controls” which received only DI water; “Slurry” which received 200 g biosolid slurry only; “Slurry+AgNPs”, which received 200 g biosolids and 9.9 mg AgNPs; and “Slurry+AgNO_3_”, which received 200 g biosolids and 44 mg Ag as AgNO_3_. The biosolids in the Slurry treatment samples were homogenized on the day of treatment with a milkshake maker (DrinkMaster Drink Mixer, Hamilton Beach, Southern Pines, USA) for 1 min just prior to slurry application, then promptly added to the soil surface of the mesocosms with care taken not to apply slurry to the foliage. The only difference for the Slurry+AgNPs and Slurry+AgNO_3_ treatments from the Slurry treatments was that the Ag was added during homogenization with the milkshake maker. Containers used for holding water or slurries were rinsed with 500 ml well water, which then was added to the soil surface.

For the separate batch assay for generation of samples that had a high Ag concentration yielding a strong enough signal to be analyzed by EXAFS (Figure 3B), a slurry was prepared as for the mesocosm experiment. Then, to a subsample of biosolids (5 g dry weight equivalent) 10.8 ml of 250 mg Ag L^−1^ stock of AgNPs was added, shaken at 120 rpm for 15 minutes, frozen in liquid nitrogen, and freeze dried.

### Sampling

We sampled the top 1 cm of surface soil using three replicate 1 cm diameter cores immediately before treatment (Day 0), and 1 and 50 days after treatment application. These samples were preserved for molecular and enzymatic analyses by freezing at −80 °C. We destructively harvested all aboveground biomass on Day 50, and subsequently removed five 5 cm diameter x 15 cm deep soil cores from each mesocosm to estimate root and microbial biomass, as well as Ag content for the soil depth increments 0–1, 1–5 and 5–10 cm.

To sample CO_2_, CH_4_, and N_2_O emissions from soil, we used 10 cm gas collars installed to 10 cm depth placed in the one space in the 4 × 4 planting grid which was left empty at planting. Caps were installed on the gas collars immediately prior to gas collection, and trace gases were allowed to accumulate. Gases were sampled every 20 minutes for one hour after capping the collars by withdrawing 10 ml of gas from the headspace, and transferring that to 9 ml evacuated serum vials.

In order to obtain an index of available NH_4_^+^, NO_3_^−^, and ortho-phosphate throughout the course of this experiment, Plant Root Simulator (PRS^TM^) probes were installed (17.5 cm^2^ area per probe; Western Ag Innovations, Saskatoon, Canada), with two anion and two cation probes installed per mesocosm between the 1^st^ and 2^nd^, or 3^rd^ and 4^th^ rows of plants. PRS^TM^-probes were installed for four time periods: one pre-experiment period (8/5/2011 to 8/26/2009), and three post-treatment time intervals (8/26/2011 to 9/16/2011, 9/16/2009 to 10/7/2009, and 10/07/2009 to 10/15/2009). Probes were rinsed in the field when removed, and fresh probes were installed in their place. Sampled probes were brought back to the lab in polyethylene bags, and were then extracted with 0.5M HCl for later analysis

### Sample processing and analysis

Soil core increments were sieved (2 mm opening) to remove rocks and roots, and homogenize samples. Subsamples were also used for microbial biomass. A separate subsample of soil from each was dried and ground for Ag analysis. Soils were microwave-digested using EPA method 3052 [35]. All silver concentrations were measured using ICP-MS (Agilent 7500CX, Santa Clara, CA, USA) as previously described[24]. The method detection limit varied by sample due to differences in sample mass. The average method detection limits were 0.066, 0.037, 0.030, 0.014, and 0.023 mg Ag/kg for 0–1 cm roots, 1–5 cm roots, 5–10 cm roots, shoots, and soils, respectively.

The aboveground plant biomass was split by species in the field, while roots were split by depth increment, not species. All biomass was then oven dried and weighed, and subsamples were ground for biomass Ag analysis. To prepare plant samples for Ag analyses, samples were digested using a modification of published methods [36] using sequential digestion of ∼0.1 g of plant material in 2 ml of concentrated HNO_3_ and 0.5 ml of 30% H_2_O_2_ heated to 70 °C for 1 hour, and 6 ml concentrated HCl at 70 °C for 1 hour [36], [37].

The fate of Ag in the soil was investigated beyond just concentration by using X-ray absorption spectroscopy (XAS) and transmission electron microscopy (TEM). The extended x-ray absorption fine structure (EXAFS) region was analyzed with linear combination fitting (LCF), with EXAFS spectra collected at the Stanford Synchrotron Radiation Laboratory (SSRL) BL4-1. Both the EXAFS data collection and LCF analysis have been described in detail in previously published studies.

For detailed TEM analysis, we used an FEI Titan 80–300 field emission TEM operating at 200 kV. The microscope is equipped with an energy dispersive X-ray spectrometer (EDAX r-TEM) for chemical analysis. The chemistry of individual particles of interest was examined with a lateral spatial resolution of approximately 1 nm using the Scanning TEM mode. High-resolution TEM analyses were used to examine the crystal structure of the particles of interest.

To measure microbial biomass, we used a standard method, substrate induced respiration (SIR) biomass [38]. Briefly, 4 g of soil was weighed into 40 ml I-chem vials into which was added 10 ml of yeast extract (12 mg mL^−1^; BD Difco, Sparks, USA). Following yeast addition, samples were shaken on an orbital shaker at 120 rpm and CO_2_ concentration was measured after 30 min, 2, and 4 hours using an infrared gas analyzer (Li-Cor 6400, Lincoln, USA).

Trace gas fluxes from field soils were determined by analyzing gas samples from the mesocosm gas collars on a Shimadzu GC with FID and ECD detectors (Shimadzu, Columbia, USA). The rate of accumulation over time of CO_2_, CH_4_, and N_2_O were first examined for linearity, with data with an r^2^<0.7 being set to zero if the nonlinearity was due to no slope. Slopes were then converted into mg of C or N m^−2^ hour^−1^.

Extracellular enzyme activity was determined for samples taken in surface 0–1 cm soils sampled at the end of the experiment (October 14, 2009). Activity of the following three enzymes was measured using published methods[39]: aryl-sulfatase, leucine aminopeptidase, and alkaline phosphatase. Samples were frozen prior to analysis, which may influence the absolute rates of this potential assay, but should have affected all soils in the same way leaving comparisons between treatments valid[40].

For each soil sampling time-point and treatment, total DNA was extracted from four individual 0.25 g replicates using the PowerSoil® DNA Isolation Kit (MoBio Laboratories, Solana Beach, USA). All DNA extractions were performed following the manufacturer's protocol with slight modification. The manufacturer's bead beating step was extended to 15 min. Following extraction, DNA was stored in elution buffer at −20 °C until further use. DNA quality and concentration was verified using an ND-1000 NanoDrop spectrophotometer (NanoDrop Technologies, Wilmington, USA). DNA concentrations ranged from 5 to 20 ng/µL and quality was verified to ensure minimal phenolic or protein contamination.

PCR amplification of bacterial 16S SSU rDNA was performed following the protocol described in Lukow et al. [41] with slight modification. The forward primer (27F) was fluorescently labeled with 6-carboxyfluorescein. One uL of template DNA was used in a 100 µL reaction. Bovine serum albumin (10 µg) was added to limit primer dimer formation and humic acid interference. The number of cycles was extended to 40 to increase amplification. The presence of PCR amplicons of the correct length was verified by visualization on a 1% agarose gel containing 0.1% ethidium bromide. PCR amplicons were purified using Qiagen PCR Purification Kits (Qiagen, Hilden, Germany) following the manufacturer's protocol. Samples were eluted to a final volume of 50 µL in elution buffer. Final PCR product concentrations and purity were verified as described above.

Restriction enzyme digests were performed using previously described methods [41]. Purified PCR product was digested with HaeII and MspI (New England Bio-labs Inc., Beverly, USA). The mixture was incubated at 37 °C for 2 hours, followed by 15 min heating at 65 °C for enzymatic inactivation. All samples were desalted by spin column filtration, and fragment analysis was carried out using an Applied Biosystems 3100 capillary sequencer (Foster City, USA) with POP6 polymer and ROX-labeled MapMarker 1000 size standards (BioVentures, Inc., Murfreesboro, USA). All analyses were carried out at the Duke University DNA Analysis Facility (Durham, USA) following standard procedures.

### Statistics

To examine data for treatment-level effects, data were compared using analysis of variance (ANOVA). Data were first tested for normality using the Shapiro-Wilk test, and then for equal variance. If data were found to be normal and have equal variance, data were analyzed using ANOVA with Holm-Sidak post-hoc comparisons. For data that were either non-normal or had unequal variance, Kruskal-Wallis ANOVA by ranks was used, with Student-Newman-Keuls post-hoc tests. These analyses were all conducted using SigmaStat 4 (Systat Software Inc., San Jose, USA)[42]. For all ANOVA tests, we used a group-wise α = 0.05.

Restriction fragment profiles (from T-RFLP digests) were visualized using GeneScan v3.7.1 software (Applied Biosystems, Foster City, USA). Raw fragment data were imported into T-REX [43] which was then used to select true peaks in all profiles, align restriction fragments, and then create presence/absence matrices for all restriction fragments. All restriction fragments smaller than 50 base pairs were excluded to ensure primer dimer fragments were excluded, and remaining peaks were also excluded if they did not meet a peak height threshold of 50 relative fluorescent units. Operational taxonomic unit (OTU) richness (total number of taxa) of T-RFLP profiles was determined for all profiles and compared to control mesocosms using a two tailed student's t-test (p<0.05). PC-ORD[44] was used to generate ordinations from presence/absence data by nonmetric multidimensional scaling (NMS) with Sørenson distance using the medium auto-pilot function [45]. Each point on the ordination represents an average of the six experimental replicates for a given day/treatment combination, with points closer together being more similar than those that are farther apart. Ordination plots were analyzed with analysis of similarity (ANOSIM) performed using the PAST statistical software to determine if ordination clusters by treatment were statistically similar [46]. The null hypothesis assumes that there are no differences between community composition at given sampling dates [47], and is assessed by examining the R value and p-value. The R-value indicates how closely related each replicate within a treatment is to other replicates within that treatment, as opposed to replicates in other treatments, while p-values suggest the probability that the R-value is due to random chance as opposed to a treatment effect. R-values range from −1 to 1, and an R-value between 0 and 1 indicates that the treatments are more similar to their own replicates than those from other treatments, while an R-value of −1 to 0 indicates the replicates in a given treatment are more similar to replicates from other treatments than replicates from their own treatment.

## Supporting Information

Figure S1 **TEM image of TiO2 nanoparticle aggregate with associated Ag. A** TEM image of TiO_2_ nanoparticle aggregate from surface soils in Slurry+AgNPs treatment, and **B** EDX spectra of area on aggregate highlighted with a white circle in **A** showing Ag associated with TiO_2_-nanoparticle aggregate (adapted from Figure 5 in reference 25, http://dx.doi.org/10.1039/C2EM10809G, and reproduced by permission of The Royal Society of Chemistry)
(TIF)

Figure S2 **Total aboveground plant biomass by species, and belowground root biomass by depth.** Shared letters denote no significant difference at p<0.05 between treatments for either total aboveground or belowground biomass
(TIF)

Figure S3 **Microbial biomass in soils.** **A** 0–1 cm soils, **B** 1–5 cm soils, and **C** 5–10 cm soils. Shared letters denote no significant difference at p<0.05 between treatments, and error bars are standard error of the mean (n = 6)
(TIF)

Figure S4 **Plant Root Simulator^TM^ Resin available NO_3_^−^ and phosphate.** Shared letters denote no significant difference at p<0.05 between treatments, and error bars are standard error of the mean (n = 6)
(TIF)

Table S1 **Plant biomass, Ag concentration, and Ag content by mesocosm.** Shared letters denote no significant difference at p<0.05 between treatments within a plant species and sampling date, and error terms are one standard deviation
(DOCX)

Table S2 **Photosynthetic rates for the four plant species measured.** Shared letters denote no significant difference at p<0.05 between treatments within a plant species and sampling date, and error terms are standard error of the mean (n = 8 measurements from plants from the six replicate mesocosms per treatment)
(DOCX)
