# Supplementary figures and images for: Low Concentrations of Silver Nanoparticles in Biosolids Cause Adverse Ecosystem Responses under Realistic Field Scenario
Source: PLoS One. 2013 Feb 27;8(2):e57189. doi: 10.1371/journal.pone.0057189 (PMC3584129; doi:10.1371/journal.pone.0057189)

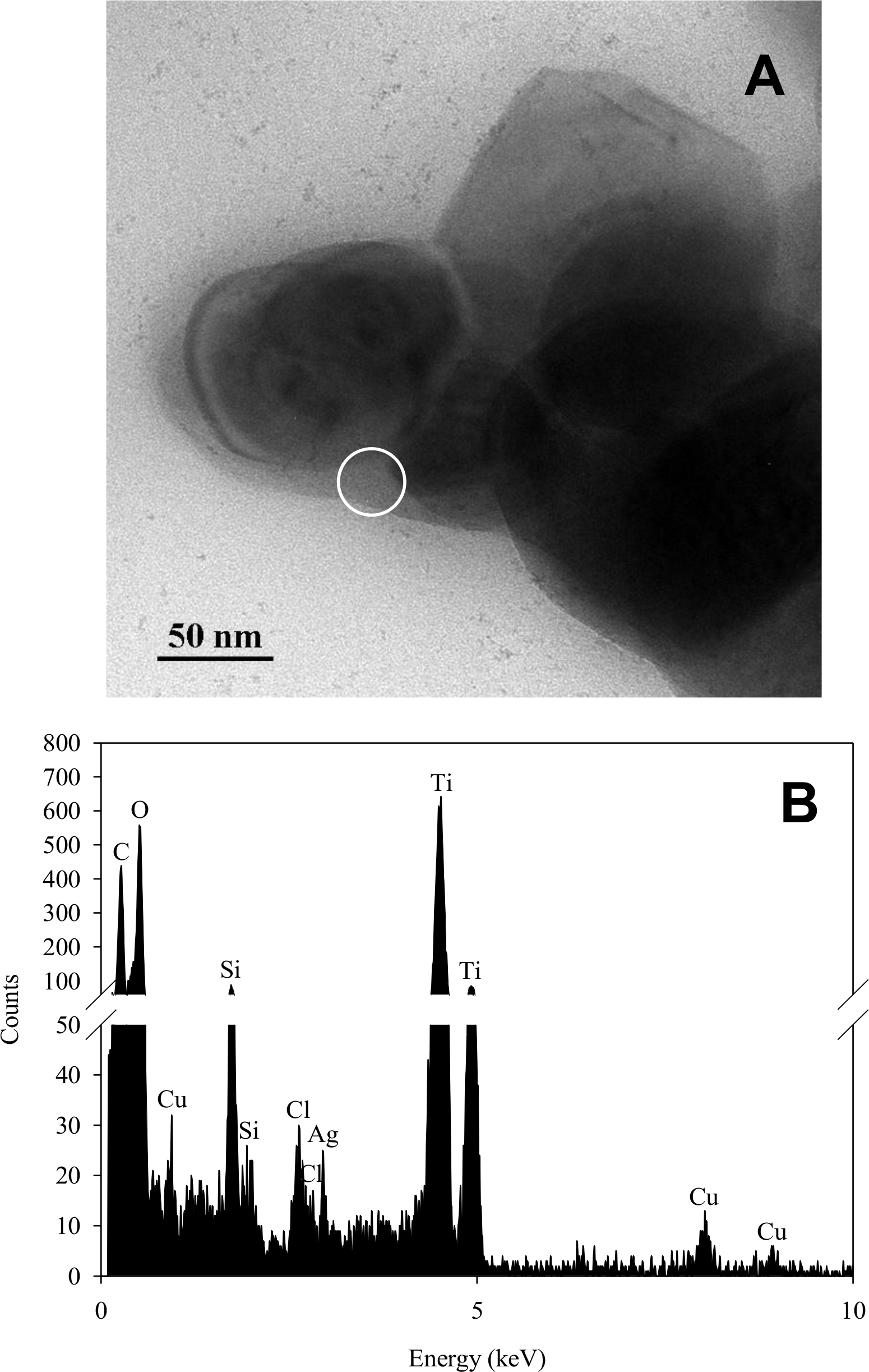

Supplement: Figure S1 — TEM image of TiO2 nanoparticle aggregate with associated Ag. A TEM image of TiO2 nanoparticle aggregate from surface soils in Slurry+AgNPs treatment, and B EDX spectra of area on aggregate highlighted with a white circle in A showing Ag associated with TiO2-nanoparticle aggregate (adapted from Figure 5 in reference 25, http://dx.doi.org/10.1039/C2EM10809G, and reproduced by permission of The Royal Society of Chemistry) (TIF) [file pone.0057189.s001.tif]

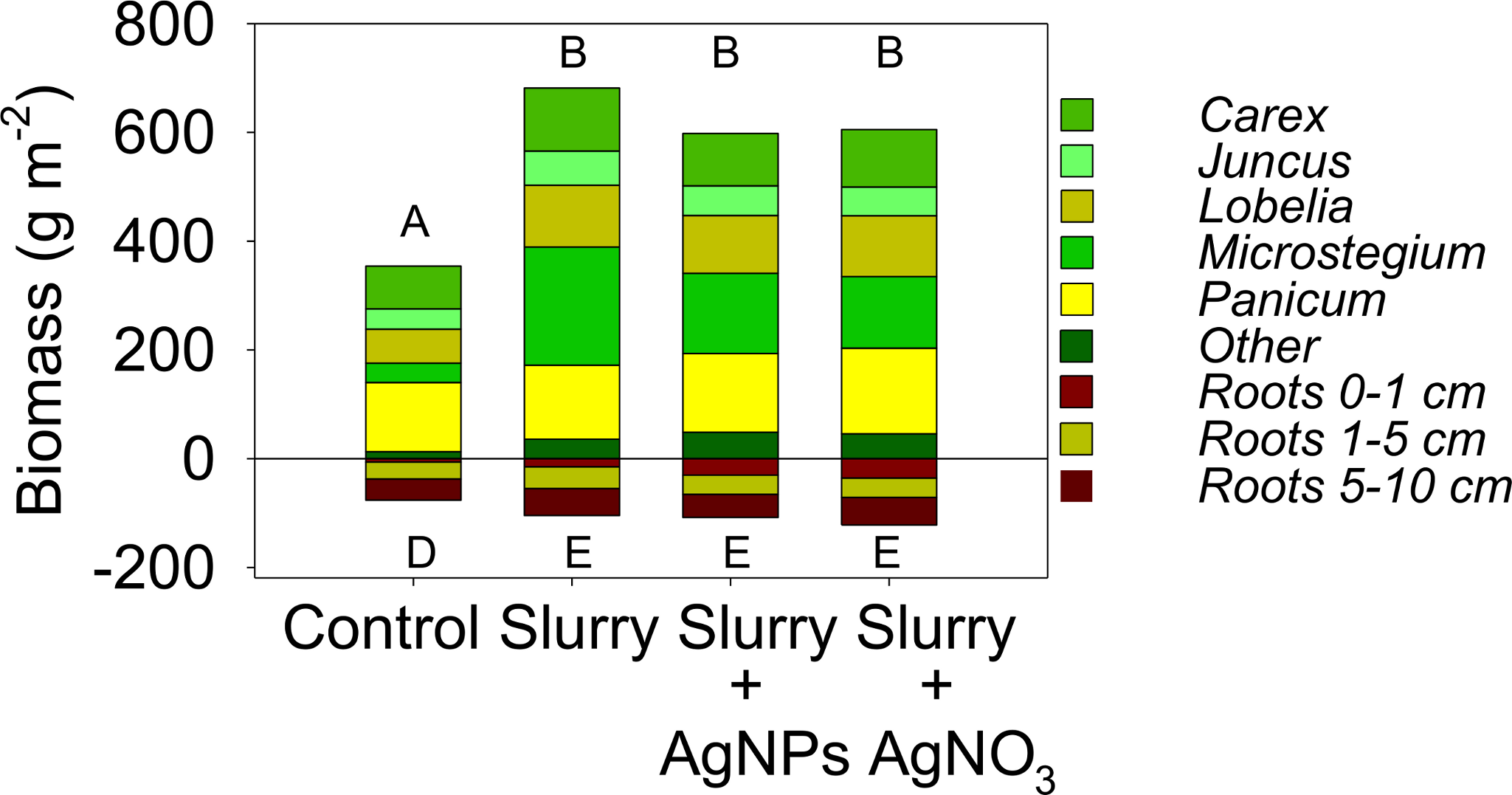

Supplement: Figure S2 — Total aboveground plant biomass by species, and belowground root biomass by depth. Shared letters denote no significant difference at p<0.05 between treatments for either total aboveground or belowground biomass (TIF) [file pone.0057189.s002.tif]

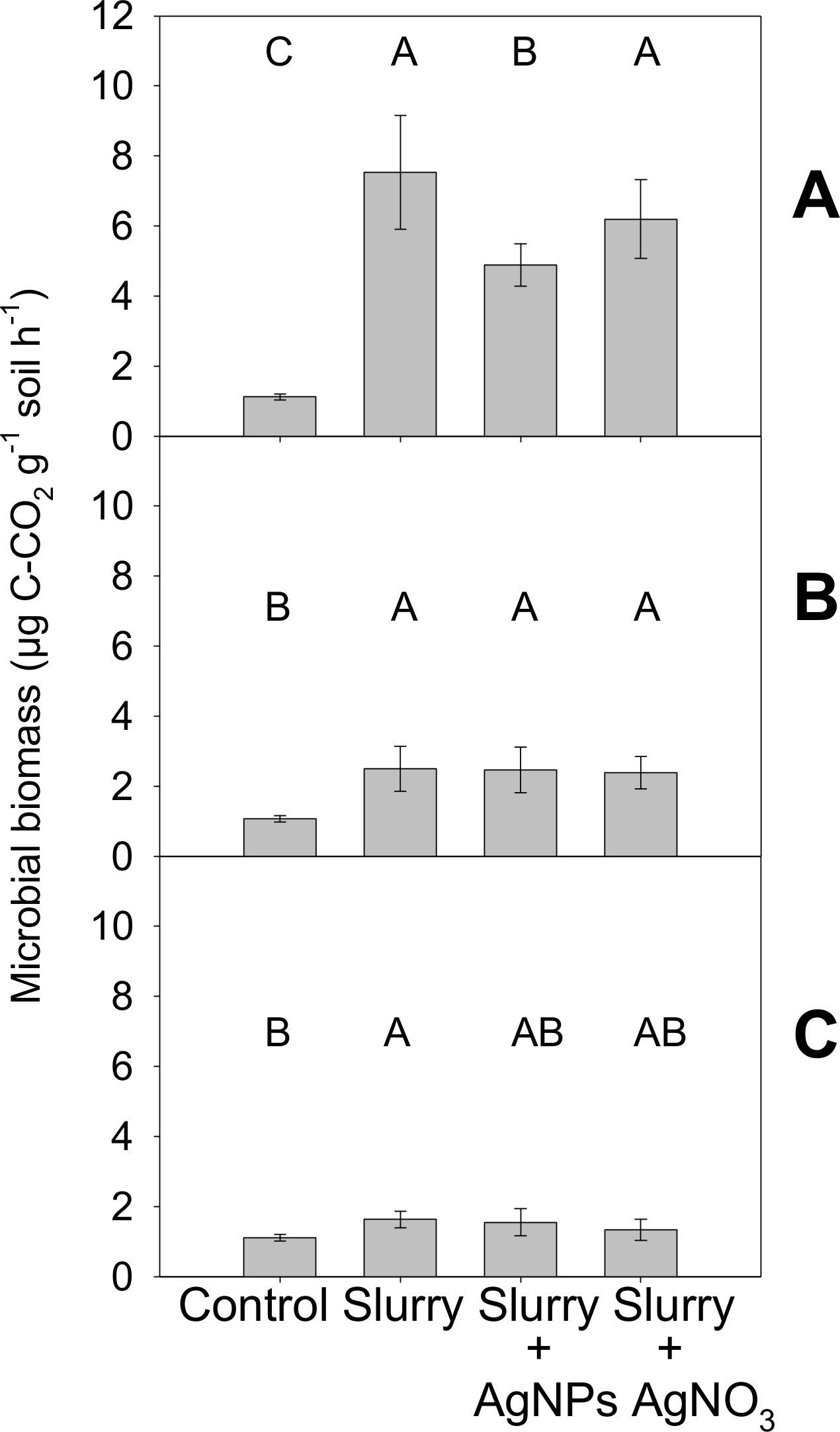

Supplement: Figure S3 — Microbial biomass in soils. A 0–1 cm soils, B 1–5 cm soils, and C 5–10 cm soils. Shared letters denote no significant difference at p<0.05 between treatments, and error bars are standard error of the mean (n = 6) (TIF) [file pone.0057189.s003.tif]

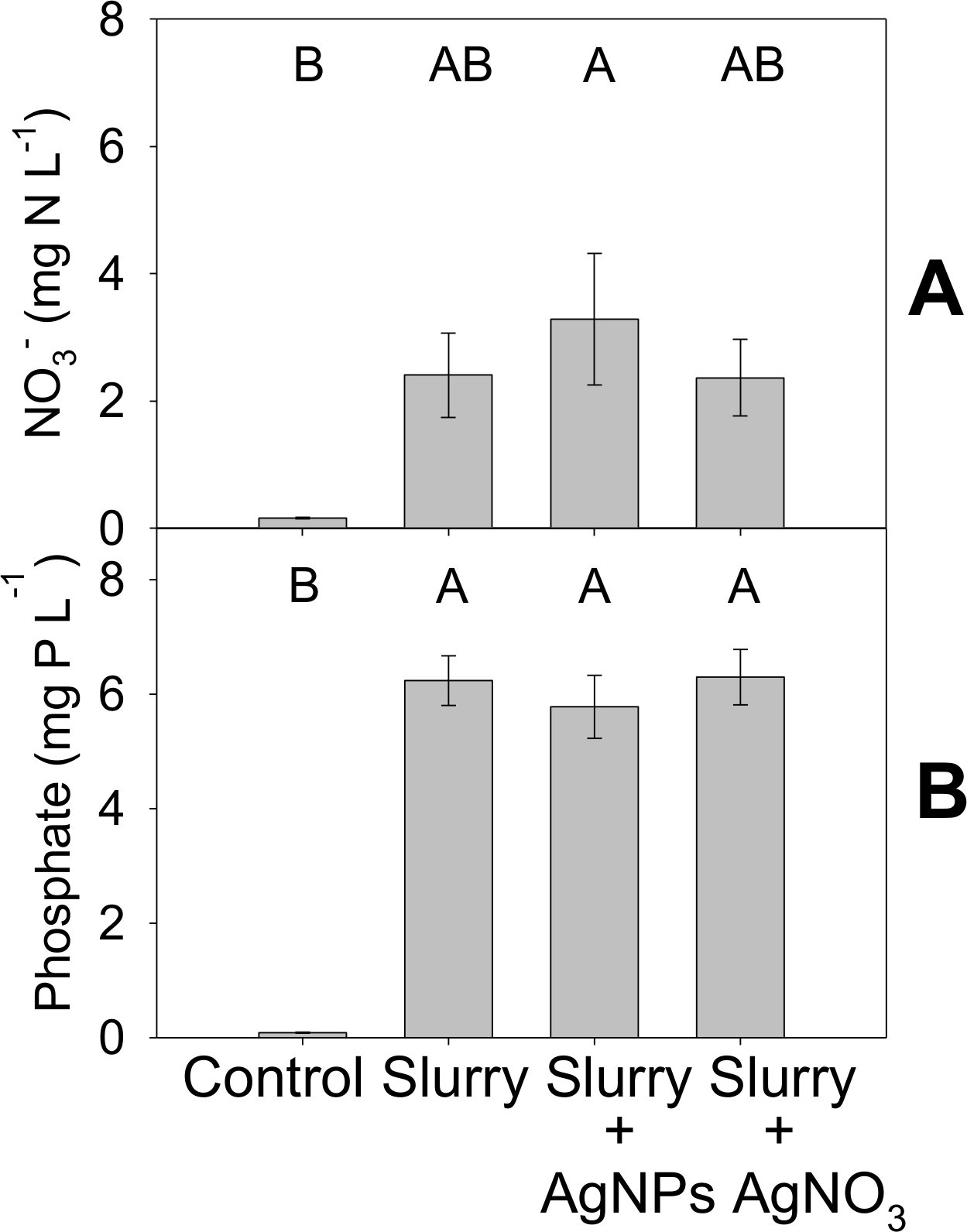

Supplement: Figure S4 — Plant Root SimulatorTM Resin available NO3− and phosphate. Shared letters denote no significant difference at p<0.05 between treatments, and error bars are standard error of the mean (n = 6) (TIF) [file pone.0057189.s004.tif]
