# Supplementary material for: Low Concentrations of Silver Nanoparticles in Biosolids Cause Adverse Ecosystem Responses under Realistic Field Scenario
Source: PLoS One. 2013 Feb 27;8(2):e57189. doi: 10.1371/journal.pone.0057189 (PMC3584129; doi:10.1371/journal.pone.0057189)
Supplement: Table S1 — Plant biomass, Ag concentration, and Ag content by mesocosm. Shared letters denote no significant difference at p<0.05 between treatments within a plant species and sampling date, and error terms are one standard deviation (DOCX) [file pone.0057189.s005.docx]

**Supplementary Table 1: Plant biomass, Ag concentration, and Ag content by mesocosm.**

| Tissue | Treatment | Biomass (g/m2) | | | | Ag (mg/kg) | | | | Ag (µg/Mesocosm) | | | |
| --- | --- | --- | --- | --- | --- | --- | --- | --- | --- | --- | --- | --- | --- |
| *Carex* | Control | 78.7 | ± | 7.5 | A | 0.03 | ± | 0.01 | B | 0.6 | ± | 0.1 | B |
| *Carex* | Slurry | 115.8 | ± | 13.4 | A | 0.04 | ± | 0.02 | B | 1.0 | ± | 0.7 | B |
| *Carex* | Slurry+AgNPs | 96.3 | ± | 6.8 | A | 0.21 | ± | 0.11 | A | 4.6 | ± | 2.9 | A |
| *Carex* | Slurry+AgNO_3_ | 105.6 | ± | 14.0 | A | 0.34 | ± | 0.17 | A | 7.8 | ± | 3.8 | A |
| *Juncus* | Control | 37.2 | ± | 6.5 | B | 0.04 | ± | 0.01 | C | 0.3 | ± | 0.1 | D |
| *Juncus* | Slurry | 62.9 | ± | 7.4 | A | 0.04 | ± | 0.02 | C | 0.6 | ± | 0.3 | C |
| *Juncus* | Slurry+AgNPs | 54.5 | ± | 5.2 | AB | 0.10 | ± | 0.06 | B | 1.3 | ± | 0.7 | B |
| *Juncus* | Slurry+AgNO_3_ | 53.1 | ± | 3.7 | AB | 0.20 | ± | 0.08 | A | 2.4 | ± | 1.0 | A |
| *Lobelia* | Control | 62.5 | ± | 1.5 | B | 0.04 | ± | 0.04 | B | 0.6 | ± | 0.7 | C |
| *Lobelia* | Slurry | 113.5 | ± | 11.9 | A | 0.04 | ± | 0.02 | B | 1.0 | ± | 0.8 | C |
| *Lobelia* | Slurry+AgNPs | 106.2 | ± | 8.1 | A | 0.26 | ± | 0.34 | B | 6.8 | ± | 9.9 | B |
| *Lobelia* | Slurry+AgNO_3_ | 111.7 | ± | 7.5 | A | 1.74 | ± | 1.38 | A | 43.2 | ± | 36.5 | A |
| *Microstegium* | Control | 35.6 | ± | 6.8 | C | 0.05 | ± | 0.04 | B | 0.8 | ± | 0.9 | B |
| *Microstegium* | Slurry | 217.6 | ± | 30.3 | A | 0.04 | ± | 0.03 | B | 1.2 | ± | 0.8 | AB |
| *Microstegium* | Slurry+AgNPs | 147.6 | ± | 14.4 | B | 0.34 | ± | 0.40 | AB | 10.1 | ± | 14.1 | AB |
| *Microstegium* | Slurry+AgNO_3_ | 131.6 | ± | 17.8 | B | 3.75 | ± | 5.63 | A | 81.4 | ± | 81.1 | A |
| *Panicum* | Control | 127.1 | ± | 6.7 | A | 0.02 | ± | 0.01 | C | 0.6 | ± | 0.2 | C |
| *Panicum* | Slurry | 135.9 | ± | 10.7 | A | 0.02 | ± | 0.01 | C | 0.4 | ± | 0.2 | C |
| *Panicum* | Slurry+AgNPs | 144.8 | ± | 10.8 | A | 0.04 | ± | 0.02 | B | 1.2 | ± | 0.7 | B |
| *Panicum* | Slurry+AgNO_3_ | 157.8 | ± | 11.6 | A | 0.10 | ± | 0.07 | A | 3.3 | ± | 2.6 | A |
| Other | Control | 13.0 | ± | 3.0 | B | 0.04 | ± | 0.03 | B | 0.1 | ± | 0.1 | C |
| Other | Slurry | 35.9 | ± | 9.7 | A | 0.08 | ± | 0.07 | B | 0.9 | ± | 0.7 | B |
| Other | Slurry+AgNPs | 48.7 | ± | 7.1 | A | 0.43 | ± | 0.25 | A | 4.3 | ± | 3.7 | A |
| Other | Slurry+AgNO_3_ | 45.7 | ± | 12.2 | A | 4.05 | ± | 7.60 | A | 35.0 | ± | 63.9 | A |
| Roots 0-1cm | Control | 6.4 | ± | 1.5 | C | 0.30 | ± | 0.14 | C | 0.4 | ± | 0.1 | D |
| Roots 0-1cm | Slurry | 15.2 | ± | 2.9 | B | 0.56 | ± | 0.19 | B | 1.9 | ± | 1.2 | C |
| Roots 0-1cm | Slurry+AgNPs | 30.1 | ± | 8.0 | A | 3.07 | ± | 1.66 | A | 24.5 | ± | 25.4 | B |
| Roots 0-1cm | Slurry+AgNO_3_ | 35.9 | ± | 6.3 | A | 12.87 | ± | 10.40 | A | 128.8 | ± | 151.0 | A |
| Roots 1-5cm | Control | 31.2 | ± | 8.0 | A | 0.17 | ± | 0.07 | B | 1.0 | ± | 0.4 | B |
| Roots 1-5cm | Slurry | 39.8 | ± | 8.0 | A | 0.30 | ± | 0.41 | B | 3.0 | ± | 4.8 | B |
| Roots 1-5cm | Slurry+AgNPs | 35.5 | ± | 3.1 | A | 0.35 | ± | 0.16 | B | 2.7 | ± | 1.4 | B |
| Roots 1-5cm | Slurry+AgNO_3_ | 35.5 | ± | 5.2 | A | 3.05 | ± | 2.87 | A | 20.2 | ± | 15.6 | A |
| Roots 5-10cm | Control | 38.7 | ± | 6.4 | A | 0.13 | ± | 0.05 | A | 1.0 | ± | 0.3 | A |
| Roots 5-10cm | Slurry | 49.4 | ± | 8.6 | A | 0.17 | ± | 0.18 | A | 1.4 | ± | 0.8 | A |
| Roots 5-10cm | Slurry+AgNPs | 42.3 | ± | 10.0 | A | 0.32 | ± | 0.28 | A | 1.9 | ± | 1.0 | A |
| Roots 5-10cm | Slurry+AgNO_3_ | 50.5 | ± | 7.6 | A | 0.22 | ± | 0.21 | A | 2.5 | ± | 2.7 | A |

Letters denote differences between treatments within a plant species and sampling date, and error terms are standard deviation (n = 6)
