# Supplementary material for: Low Concentrations of Silver Nanoparticles in Biosolids Cause Adverse Ecosystem Responses under Realistic Field Scenario
Source: PLoS One. 2013 Feb 27;8(2):e57189. doi: 10.1371/journal.pone.0057189 (PMC3584129; doi:10.1371/journal.pone.0057189)
Supplement: Table S2 — Photosynthetic rates for the four plant species measured. Shared letters denote no significant difference at p<0.05 between treatments within a plant species and sampling date, and error terms are standard error of the mean (n = 8 measurements from plants from the six replicate mesocosms per treatment) (DOCX) [file pone.0057189.s006.docx]

**Supplementary Table 2: Photosynthetic rates for the four plant species measured.**

|  |  | Photosynthesis (µmol CO2 m^-2^ leaf area^-1^) | | | | | | | |
| --- | --- | --- | --- | --- | --- | --- | --- | --- | --- |
| Species | Treatment | Day 8 | | | | Day 30 | | | |
| *Carex* | Control | 7.9 | ± | 0.8 | A | 4.7 | ± | 1.0 | A |
| *Carex* | Slurry | 15.2 | ± | 3.0 | A | 9.3 | ± | 1.5 | A |
| *Carex* | Slurry+AgNPs | 12.4 | ± | 0.9 | A | 7.6 | ± | 0.9 | A |
| *Carex* | Slurry+AgNO_3_ | 12.1 | ± | 0.8 | A | 12.2 | ± | 1.1 | A |
| *Lobelia* | Control | 14.2 | ± | 1.1 | A | 10.9 | ± | 1.3 | B |
| *Lobelia* | Slurry | 19.3 | ± | 1.3 | A | 20.4 | ± | 0.2 | A |
| *Lobelia* | Slurry+AgNPs | 18.3 | ± | 0.6 | A | 19.4 | ± | 0.9 | A |
| *Lobelia* | Slurry+AgNO_3_ | 19.3 | ± | 1.2 | A | 18.6 | ± | 1.3 | A |
| *Microstegium* | Control | 8.6 | ± | 1.2 | B | 5.7 | ± | 0.8 | A |
| *Microstegium* | Slurry | 27.5 | ± | 4.1 | A | 26.2 | ± | 3.3 | A |
| *Microstegium* | Slurry+AgNPs | 30.3 | ± | 4.1 | A | 30.0 | ± | 3.6 | A |
| *Microstegium* | Slurry+AgNO_3_ | 13.6 | ± | 2.5 | AB | 22.2 | ± | 6.1 | A |
| *Panicum* | Control | 21.5 | ± | 1.4 | A | 29.7 | ± | 2.3 | A |
| *Panicum* | Slurry | 19.6 | ± | 3.1 | A | 27.8 | ± | 1.4 | A |
| *Panicum* | Slurry+AgNPs | 22.2 | ± | 2.0 | A | 35.1 | ± | 2.9 | A |
| *Panicum* | Slurry+AgNO_3_ | 29.0 | ± | 2.6 | A | 35.4 | ± | 2.1 | A |

Letters denote significant differences at p< 0.05 between treatments within a plant species and sampling date, and error terms are standard error of the mean (n = 8 measurements from plants from the six replicate mesocosms per treatment)
